# Mast cells express IL17A, IL17F and RORC, are activated and persist with IL-17 production in resolved skin of patients with chronic plaque-type psoriasis

**DOI:** 10.21203/rs.3.rs-3958361/v1

**Published:** 2024-02-16

**Authors:** Theresa Benezeder, Natalie Bordag, Johannes Woltsche, Andrea Teufelberger, Isabella Perchthaler, Wolfgang Weger, Wolfgang Salmhofer, Alexandra Gruber-Wackernagel, Clemens Painsi, Qian Zhan, Amin El-Heliebi, Magda Babina, Rachael Clark, Peter Wolf

**Affiliations:** Department of Dermatology and Venereology, Medical University of Graz; Department of Dermatology and Venereology, Medical University of Graz; Department of Dermatology and Venereology, Medical University of Graz; Department of Dermatology and Venereology, Medical University of Graz; Department of Dermatology and Venereology, Medical University of Graz; Department of Dermatology and Venereology, Medical University of Graz; Department of Dermatology and Venereology, Medical University of Graz; Department of Dermatology and Venereology, Medical University of Graz; State Hospital Klagenfurt; Department of Dermatology, Brigham and Women’s Hospital, Harvard Medical School; Division of Cell Biology, Histology and Embryology, Gottfried Schatz Research Center, Medical University of Graz; Institute of Allergology, Charite-Universitatsmedizin Berlin; Harvard Medical School/Brigham and Women’s Hosptial; Department of Dermatology and Venereology, Medical University of Graz, Graz, Austria

## Abstract

Little is known about IL-17 expression in psoriasis and the actual cellular source of IL-17 remains incompletely defined. We show that high numbers of IL-17 + mast cells persisted in resolved lesions after treatment (anti-IL-17A, anti-IL-23, UVB or topical dithranol) and correlated inversely with the time span in remission. IL-17 + mast cells were found in T cell-rich areas and often close to resident memory T cells (T_rm_) in active psoriasis and resolved lesional skin. Digital cytometry by deconvolution of RNA-seq data showed that activated mast cells were increased in psoriatic skin, while resting mast cells were almost absent and both returned to normal levels after treatment. When primary human skin mast cells were stimulated with T cell cytokines (TNFα, IL-22 and IFNγ), they responded by releasing more IL-17A, as measured by ELISA. *In situ* mRNA detection using padlock probes specific for transcript variants of *IL17A, IL17F*, and *RORC* (encoding the Th17 transcription factor RORγt) revealed positive mRNA signals for *IL17A, IL17F*, and RORCin tryptase + cells, demonstrating that mast cells have the transcriptional machinery to actively produce IL-17. Mast cells thus belong to the center of the IL-23/IL-17 axis and high numbers of IL-17 + mast cells predict an earlier disease recurrence.

## Introduction

Psoriasis is a common chronic inflammatory skin disease, in which patients present with erythematous scaly skin lesions. Although lesions tend to occur at predilection sites such as the knees, elbows and sacral area, they can occur all over the body (1–3). The main histologic features of plaque-type psoriasis include epidermal hyperproliferation and an inflammatory infiltrate of leukocytes in the dermis (4, 5). Even in mild forms of the disease, the burden on patients is substantial (6). In the last decade, major advances have been made in the systemic treatment of psoriasis. Targeted therapy with antibodies against IL-23 or IL-17 shows high efficacy and allows the clinical clearance of psoriatic lesions in a large number of patients (7–10). Currently, T-helper cells, and particularly Th17 cells, are thought to be the major source of the proinflammatory cytokine IL-17 in psoriasis (5, 11, 12). However, recent studies have indicated that innate immune cells such as type 3 innate lymphoid cells (ILC3s), natural killer (NK) cells, neutrophils and mast cells (MCs) also produce IL-17 in psoriasis (13–16). Nevertheless, little is known about IL-17 expression in active psoriasis and in resolved skin lesions after treatment. In addition, the actual cellular source of IL-17 remains unclear; for example, Noordenbos et al. suggested that mast cells do not produce IL-17A themselves, but are able to capture, store and release bioactive IL-17A (17). In addition to their role in IgE-mediated allergic reactions (18), mast cells can be activated by many different triggers and form an important component of innate immunity, where they play roles in many other biological processes, e.g. response to pathogens, angiogenesis, or wound healing (19, 20). In recent years, it became clear that mast cells are involved in the pathogenesis of a wide spectrum of diseases, including not only mast cell-driven disorders such as urticaria or mastocytosis but also autoimmune and other inflammatory disorders and even malignant tumors (21). Their role in immunological skin diseases like atopic dermatitis, bullous pemphigoid, scleroderma, and chronic graft-versus-host disease is currently under investigation. In all these skin diseases, mast cell numbers are increased and undergo degranulation (22). Under certain circumstances, mast cells may play a protective role in the pathophysiology of several skin conditions (23, 24).

After successful therapy, psoriatic skin lesions may resolve completely clinically, but lesions often reoccur at the exact same body sites within months of treatment discontinuation (25, 26). This leads us to question which cells remaining in the skin could potentially cause the recurrence of psoriasis. In areas of clinically resolved psoriasis (postlesional skin), tissue-resident memory T cells (T_RM_) are a local source of cytokines and have the potential to create a proinflammatory environment that could cause disease recurrence (27, 28). After *ex vivo* stimulation, these cells can produce IL-17 and express *RORC*, the gene encoding RORγt (Th17 transcription factor), and *IL22* mRNA (29, 30).

Below we report our study findings, namely that mast cells express *RORC, IL17F*, and *IL17A* mRNA, are activated in psoriasis and return to a resting state in lesional skin after treatment. They reside in T cell-rich areas and persist over long periods with IL-17 production in clinically resolved lesions and thus, together with T cells, mast cells belong to the center of the IL-23/IL-17 axis underlying psoriasis pathogenesis and might play a role in psoriasis recurrence.

## Results

### Mast cells make up the majority of IL-17 + cells in psoriasis and persist after treatment

Previous studies on IL-17 expression in psoriasis focused mainly on isolated and *ex vivo* stimulated T cells to optimize the subsequent flow cytometry analysis of cytokine expression (31–33). While this concept is very useful to analyze cellular differentiation states, it is not possible to determine which cells actually contain intracellular IL-17 in psoriasis and might not reflect the actual *in vivo* setting.

**Figure 1:**
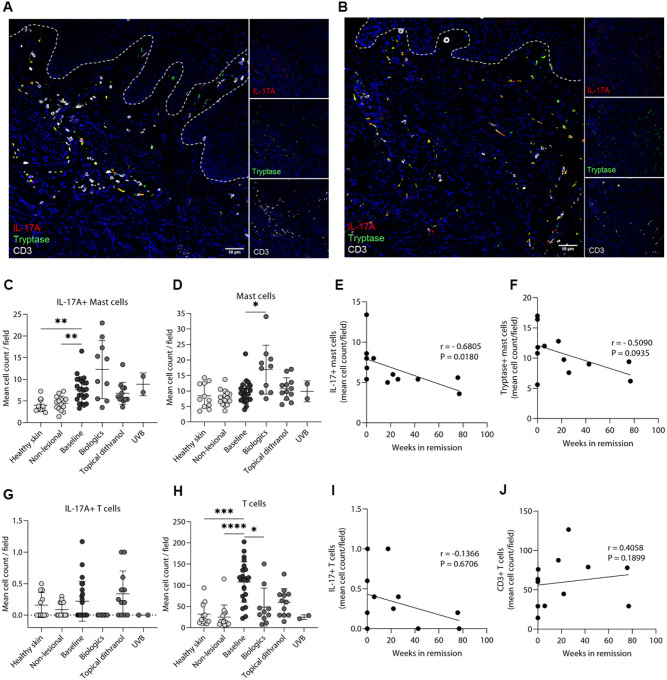
Mast cells, neutrophils and T cells are IL-17A+ at baseline, and high numbers of IL-17A+ mast cells persist in clinically resolved lesions and correlate with remission time. Representative images of immunofluorescence staining of IL-17A, tryptase, and CD3 at baseline (A) and after anti-IL-17A therapy The dotted line highlights the epidermal-dermal junction. Nuclei are stained with DAPI and shown in blue. Scale bar = 50 μm. Cell counts of IL-17A+ mast cells (C), mast cells (D), IL-17A+ T cells (G), and T cells (H) of healthy skin, non-lesional skin, psoriatic lesions at baseline, and after treatment (biologics, topical dithranol, and UVB). The Kruskal-Wallis test with a Dunn’s multiple comparisons test were used for statistics. Lines represent mean ± SD; *P < 0.05; **P < 0.01; ***P < 0.001; ****P < 0.0001. Spearman correlation analysis of IL-17+ mast cells (E), tryptase+ mast cells (F), IL-17+ T cells (I), and CD3+ T cells (J) and weeks in remission.

Therefore, we analyzed skin biopsy samples from plaque-type psoriasis lesions (n = 23), non-lesional healthy skin from psoriasis patients (n = 15), healthy skin from control subjects (n = 11) and clinically resolved psoriatic lesions after different anti-psoriatic treatments (biologics, n = 10; topical dithranol, n = 12; and UVB, n = 2). To identify and visualize IL-17 + cells, we used multiplex immunofluorescence staining for IL-17A, myeloperoxidase (MPO) for neutrophils, tryptase for mast cells, and CD3 for T cells (supplemental Fig. 1). We found that mast cells, neutrophils, and T cells are IL-17 + in psoriasis at baseline (Fig. 1A, supplemental Fig. 1). While neutrophils disappear after treatment, some IL-17 + T cells (4.7% of all IL-17 + cells) remain, but the majority of IL-17 + cells are mast cells (95.3%) (supplemental Fig. 1D). IL-17 staining largely encompassed the cytoplasm and colocalized strongly with tryptase (supplemental Fig. 2), an enzyme expressed in the secretory granules of mast cells (34). Compared to healthy and non-lesional skin, the number of IL-17 + MCs was higher in psoriasis at baseline, and high numbers of IL-17 + MCs persisted in resolved lesions after treatment (anti-IL-17A, anti-IL-23, UVB, or topical dithranol) (Fig. 1B, 1C, supplemental Fig. 3). While the number of T cells was greatly increased in psoriasis as compared to healthy and non-lesional skin and significantly decreased after treatment (Fig. 1H), the number of mast cells was not higher at baseline than the number in healthy and non-lesional skin (Fig. 1D). To our surprise, we detected very few IL-17 + T cells (Fig. 1G). We took the opportunity to employ archived skin samples of patients treated with dithranol (as an intermittent treatment) collected during a follow-up visit after treatment ended and performed a correlation analysis for the number of remaining infiltrating cells and time in remission. In this analysis, the time span in remission significantly correlated inversely with IL-17 + MC count (r = −0.68, P = 0.0180; Fig. 1E), while the overall mast cell numbers (Fig. 1F), IL-17 + T cells (Fig. 1I) and T cell counts (Fig. 1J) did not.

### IL-17 + mast cells reside in dense T cell clusters and respond to T cell cytokines with IL-17A production

**Figure 2:**
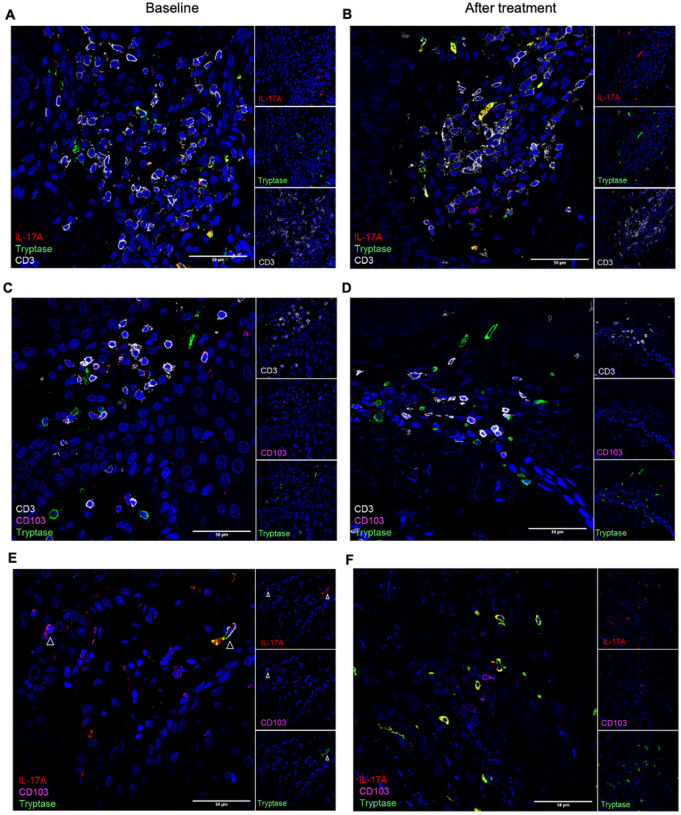
IL-17A+ mast cells (MCs) reside in dense T cell clusters at baseline (A) and after treatment (B). Mast cells can be found close to resident memory T cells (T_RM_) at baseline (C) and after treatment (D). MCs and T_RM_ are IL-17A+ at baseline (E) and MCs remain IL-17A+ after treatment, while most T_RM_ are negative for IL-17A (F). Representative immunofluorescence staining images of IL-17A, tryptase, and CD3 or CD103. Arrow heads point towards IL-17A+ T_RM_ and MCs in (E). Nuclei are stained with DAPI and shown in blue. Scale bar = 50 μm.

At baseline and after treatment, IL-17 + MCs were located in dense T cell clusters in the dermis (Fig. 2A–D). The known role of pathogenic resident memory T cells (T_RM_) in psoriasis and the localization of IL-17 + MCs in T cell-dense areas suggested that it would be useful to add CD103 as a marker for T_RM_ to our staining panel. Indeed, we saw that IL-17 + MCs are often found close to T_RM_ (Fig. 2A–D). At baseline, we located some IL-17 + CD103 + T_RM_ which often resided in the epidermis. In resolved lesions, however, T_RM_ were mostly negative for IL-17, whereas IL-17 + MCs persisted (Fig. 2E, 2F) and were located in the dermis.

**Figure 3:**
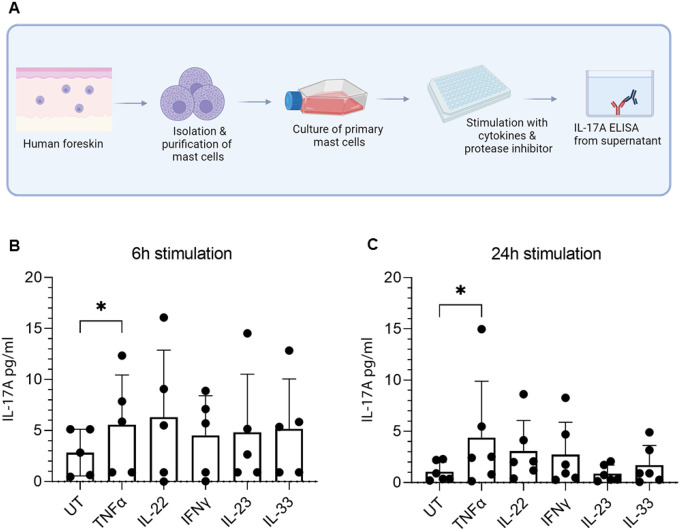
Primary mast cells respond to TNFα with IL-17A secretion. Workflow scheme: Primary mast cells were isolated from human foreskin, purified, and cultured. Mast cells were stimulated with different cytokines, and IL-17A levels were measured by ELISA. Created with BioRender.com (A). Skin-derived mast cells respond to T cell cytokines (TNFα, IL-22, and IFNγ) with increased IL-17A production after 6h (B) or 24h of stimulation with cytokines. UT = untreated. The Friedman test with a Dunn’s multiple comparisons test were used for statistics. Each data point represents one donor pool of isolated cells. Bar graphs show mean ± SD; *P < 0.05.

Next, we investigated whether the production of IL-17A by skin mast cells could be triggered *in vitro* by prominent psoriasis cytokines. We isolated MCs from human skin and stimulated them with various T-cell cytokines (IL-22, TNFα, IFNγ) in addition to the dendritic cell cytokine IL-23 and IL-33 (which is produced by hyperproliferating keratinocytes in psoriasis (35, 36)), and measured the IL-17A concentration in the supernatant by ELISA (Fig. 3). We detected an increase in IL-17A protein levels in TNFα, IL-22, IL-33, and IFNγ, and this increase became statistically significant after the cells were subjected to TNFα-treatment for 6 and 24 hours (Fig. 3B, C). Stimulation with the IgE-independent mast cell - activator Compound 48/80 (C48/80) did not increase IL-17A protein levels suggesting that specific psoriasis-relevant cytokines are needed to elicit IL-17A production (data not shown).

### Levels of activated mast cells are reduced after psoriasis treatment, while numbers of resting mast cells are increased

**Figure 4:**
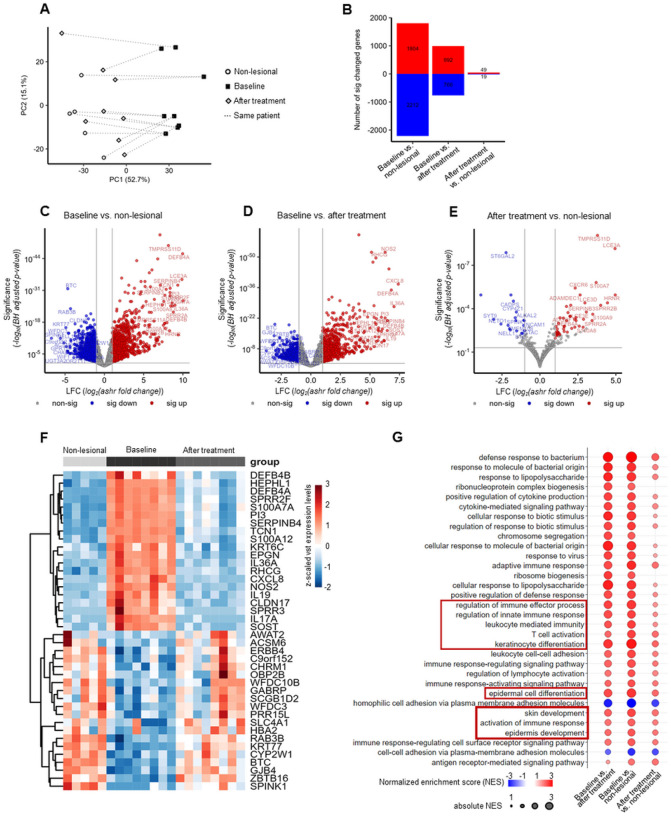
RNA-seq results of dithranol-treated psoriasis skin biopsies. PCA scores plot of the top 500 genes (highest row variance) with one dot per sample (n = 21) where nearness signifies similarity in expression. Dotted lines connect samples from the same patient (A). Bar graph (B) and volcano plots (C-E) showing differentially expressed genes (BH adjusted P < 0.05, s-value < 0.005, |LFC| > 1) for the different comparisons. Blue symbolizes significant downregulation and red upregulation (B-E). Heatmap showing top 20 significantly up- or downregulated protein-coding genes comparing baseline vs after treatment; (F). Pathway analysis showing significantly enriched gene ontology terms (G).

Previous work indicated that a small molecular scar remains after effective systemic anti-psoriatic treatment (37–39). To validate our findings on MCs after topical and systemic treatment we compared findings from dithranol-treated patients with previously published data from psoriasis patients treated with the anti-IL-17A antibody secukinumab. We have recently shown that dithranol both effectively targets keratinocytes and their crosstalk with neutrophils and downregulates IL-36-related genes after only 2–3 weeks of treatment (40, 41). However, we did not analyze clinically resolved lesions after dithranol treatment until now. Therefore, to characterize the molecular response after topical dithranol therapy, we performed bulk RNA sequencing analysis of psoriatic lesional skin at baseline (n = 8), postlesional skin after dithranol treatment (n = 8) and non-lesional skin (n = 5). As expected, we found evidence of strong transcriptional changes when comparing non-lesional skin and lesional psoriatic skin at baseline. Furthermore, we found distinct transcriptional profiles in postlesional skin as compared to baseline profiles, as demonstrated by principal component analysis (PCA) and the analysis of differentially expressed genes (DEGs) (Fig. 4A, B). Among the top 20 significantly downregulated genes after treatment were inflammatory response-related genes like *IL17A* and *IL36A*, anti-microbial peptides *(DEFB4B, DEFB4A, S100A7A, S100A12)*, chemoattractants for neutrophils *(CXCL8)* and genes involved in keratinocyte differentiation and hyperplasia *(IL19, KRT6C, SERPINB4)* (Fig. 4F). These genes are all known to be associated with psoriasis and have been shown to be significantly downregulated when exposed to treatment agents such as anti-IL-17A, anti-TNF, or anti-IL-23 biologics (13, 38, 42–47). Pathways like T cell activation, keratinocyte differentiation, epidermal cell differentiation and activation of immune response were among the most significantly enriched pathways as determined by gene set enrichment analysis (GSEA) (Fig. 4G). Some lingering molecular differences remained after dithranol treatment, although the number of differentially expressed genes identified when comparing resolved skin with non-lesional skin was low (49 genes upregulated, 19 genes downregulated, Fig. 4B, E). Genes that continued to be upregulated were those encoding anti-microbial peptides *(S100A7, S100A8, S100A9*, and *DEFB4A)* and structural genes involved in keratinocyte differentiation *(LCE3A, LCE3D, LCE3E, HRNR*, and *SERPINB3)*. In addition, we found that *CXCR6* was still upregulated in resolved skin, which is important for the generation and long-term residence of T_RM_ in peripheral tissues (Fig. 4E) (48–51).

We then downloaded and re-analyzed the RNA-seq data (GSE171012) published by Liu et al. (2022) from psoriasis patients treated with the anti-IL-17A antibody secukinumab (52) by taking the same bioinformatic approach as we used to analyze our data. Indeed, the analysis of differentially expressed genes revealed a strong concordance between the two datasets. Out of the 17,420 genes robustly quantified in both datasets, a total of 8,141 genes were regulated significantly in the same direction (i.e. either up- or downregulated), while only 31 showed controversial results, and the rest showed no significant changes in either of the two datasets (suppl. Figure 4).

In order to characterize the cellular composition in cases of active and resolved psoriasis, we employed CIBERSORTx or digital flow cytometry, a computational approach that allows identification of relative fractions of different cell types from bulk transcriptome data (53, 54).

**Figure 5:**
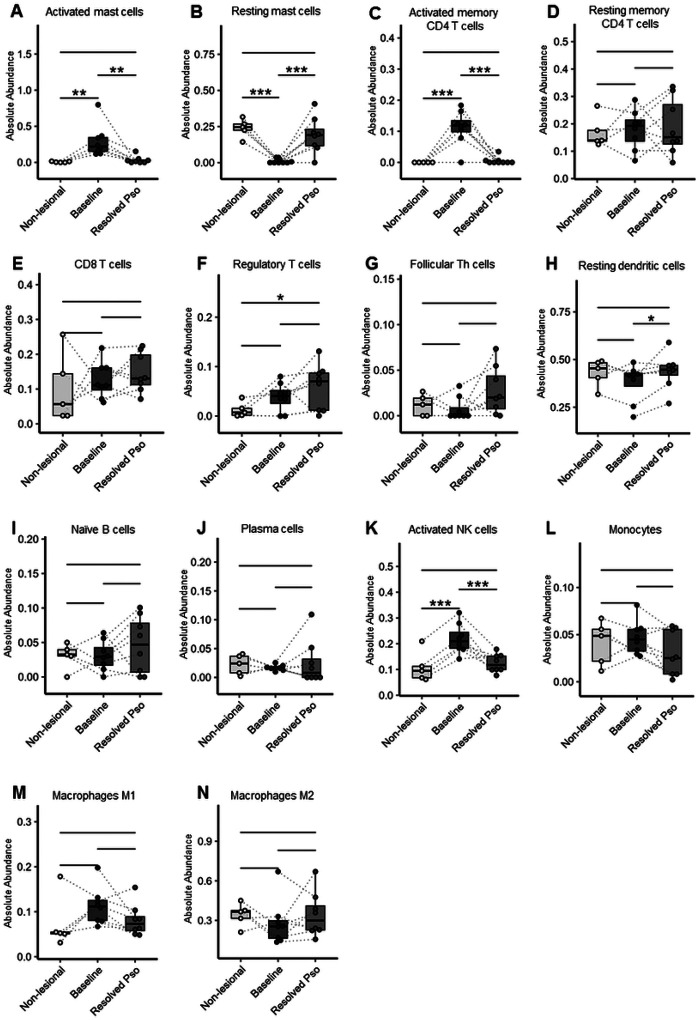
Activated mast cells are increased at baseline as compared to non-lesional skin, while resting mast cells are almost absent, and both return to normal levels after therapy (A, B), shown by digital flow cytometry via a CIBERSORTx analysis of RNA-seq data. Activated memory CD4 T cells (C) and activated NK cells (K) are increased at baseline as compared to non-lesional skin and return to normal levels after treatment. The dotted lines illustrate the paired nature of samples (from the same patient). Bars represent mean ± SD; Significance was set at *P < 0.05; **P < 0.01; ***P < 0.001 and determined by using a linear mixed effects model.

Of the 22 different immune cell types included in CIBERSORTx, we were able to detect 13 cell types in our bulk RNA-seq data set (Fig. 5). For the remaining 9 cell types (eosinophils, neutrophils, memory B cells, naïve CD4 T cells, follicular Th cells, γδT cells, resting NK cells, macrophages and activated DCs), the cell numbers or marker gene expression were too low to allow deconvolution. In addition to the identification of different cell type fractions, CIBERSORTx also allows the classification of these cells into activation states based on their expression pattern of multiple signature genes; for example, activated mast cells express *CMA1, CPA3, HOXA1, MARCH3*, and *TEC* (53, 54). We found that activated mast cell numbers were significantly higher in psoriasis at baseline than in non-lesional skin and resolved lesions after topical dithranol treatment (Fig. 5A). The opposite was true for resting mast cells, which were lowest at baseline and increased to the same level as in non-lesional sites after treatment (Fig. 5B). The number of activated memory CD4 T cells was also higher in psoriatic skin and returned to normal levels after at baseline. Furthermore, we found distinct transcriptional profiles in postlesional skin as compared to baseline profiles, as demonstrated by principal component analysis (PCA) and the analysis of differentially expressed genes (DEGs) (Fig. 4A, B). Among the top 20 significantly downregulated genes after treatment were inflammatory response-related genes like *IL17A* and *IL36A*, anti-microbial peptides *(DEFB4B, DEFB4A, S100A7A, S100A12)*, chemoattractants for neutrophils *(CXCL8)* and genes involved in keratinocyte differentiation and hyperplasia *(IL19, KRT6C, SERPINB4)* (Fig. 4F). These genes are all known to be associated with psoriasis and have been shown to be significantly downregulated when exposed to treatment agents such as anti-IL-17A, anti-TNF, or anti-IL-23 biologics (13, 38, 42–47). Pathways like T cell activation, keratinocyte differentiation, epidermal cell differentiation and activation of immune response were among the most significantly enriched pathways as determined by gene set enrichment analysis (GSEA) (Fig. 4G). Some lingering molecular differences remained after dithranol treatment, although the number of differentially expressed genes identified when comparing resolved skin with non-lesional skin was low (49 genes upregulated, 19 genes downregulated, Fig. 4B, E). Genes that continued to be upregulated were those encoding anti-microbial peptides *(S100A7, S100A8, S100A9*, and *DEFB4A)* and structural genes involved in keratinocyte differentiation *(LCE3A, LCE3D, LCE3E, HRNR*, and *SERPINB3)*. In addition, we found that *CXCR6* was still upregulated in resolved skin, which is important for the generation and long-term residence of T_RM_ in peripheral tissues (Fig. 4E) (48–51).

We then downloaded and re-analyzed the RNA-seq data (GSE171012) published by Liu et al. (2022) from psoriasis patients treated with the anti-IL-17A antibody secukinumab (52) by taking the same bioinformatic approach as we used to analyze our data. Indeed, the analysis of differentially expressed genes revealed a strong concordance between the two datasets. Out of the 17,420 genes robustly quantified in both datasets, a total of 8,141 genes were regulated significantly in the same direction (i.e. either up- or downregulated), while only 31 showed controversial results, and the rest showed no significant changes in either of the two datasets (suppl. Figure 4).

In order to characterize the cellular composition in cases of active and resolved psoriasis, we employed CIBERSORTx or digital flow cytometry, a computational approach that allows identification of relative fractions of different cell types from bulk transcriptome data (53, 54).

Of the 22 different immune cell types included in CIBERSORTx, we were able to detect 13 cell types in our bulk RNA-seq data set (Fig. 5). For the remaining 9 cell types (eosinophils, neutrophils, memory B cells, naïve CD4 T cells, follicular Th cells, γδT cells, resting NK cells, macrophages and activated DCs), the cell numbers or marker gene expression were too low to allow deconvolution. In addition to the identification of different cell type fractions, CIBERSORTx also allows the classification of these cells into activation states based on their expression pattern of multiple signature genes; for example, activated mast cells express *CMA1, CPA3, HOXA1, MARCH3*, and *TEC* (53, 54). We found that activated mast cell numbers were significantly higher in psoriasis at baseline than in non-lesional skin and resolved lesions after topical dithranol treatment (Fig. 5A). The opposite was true for resting mast cells, which were lowest at baseline and increased to the same level as in non-lesional sites after treatment (Fig. 5B). The number of activated memory CD4 T cells was also higher in psoriatic skin and returned to normal levels after treatment, while the number of resting memory CD4 T cells remained unchanged (Fig. 5C, D). In addition, we observed a slight increase in resting dendritic cells after treatment (Fig. 5H), and like MCs, activated NK cells were increased at baseline and significantly reduced after treatment (Fig. 5K).

### Mast cells express RORC, IL17F, and IL17A mRNA in psoriasis

**Figure 6:**
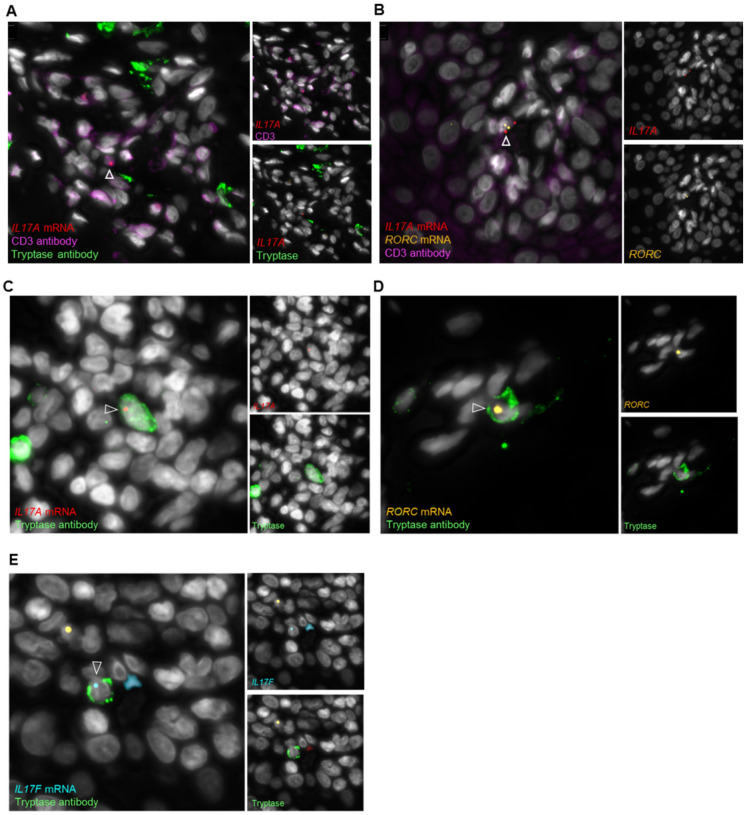
In situ mRNA detection of *IL17A, IL17F*, and *RORC in* mast cells and T cells, performed by using padlock probes in combination with immunofluorescence staining for CD3 and tryptase. Representative images showing colocalization of *IL17A* (A,B) and *RORC* (B) in CD3+ T cells and *IL17A(C), RORC* (D), and *IL17F* (E) with tryptase+ mast cells in psoriasis. Nuclei are stained with DAPI and shown in white. Arrow heads point towards positive probe signals.

Until now it has not been clear, whether mast cells capture and store IL-17 or synthesize it *de novo*. Moreover, it has been previously reported that only neutrophils and T cells have the transcriptional machinery to produce IL-17 (15, 34). Therefore, we used a highly specific *in situ* mRNA detection approach based on padlock probes targeting transcript variants of *IL17A, IL17F and RORC (which* encodes the Th17 transcription factor RORγt) in combination with immunofluorescence staining for tryptase and CD3 (Fig. 6). As expected, we found positive signals for *IL17A* and *RORC in* CD3 + T cells (Fig. 6A, B). But we were surprised to observe positive signals for *RORC, IL17A*, and *IL17F* mRNA transcripts in tryptase + mast cells (Fig. 6C–E). These results show for the first time that mast cells not only synthesize the cytokine *de novo* but also might use the same transcription factor as T cells for IL-17 production.

## Discussion

**Figure 7:**
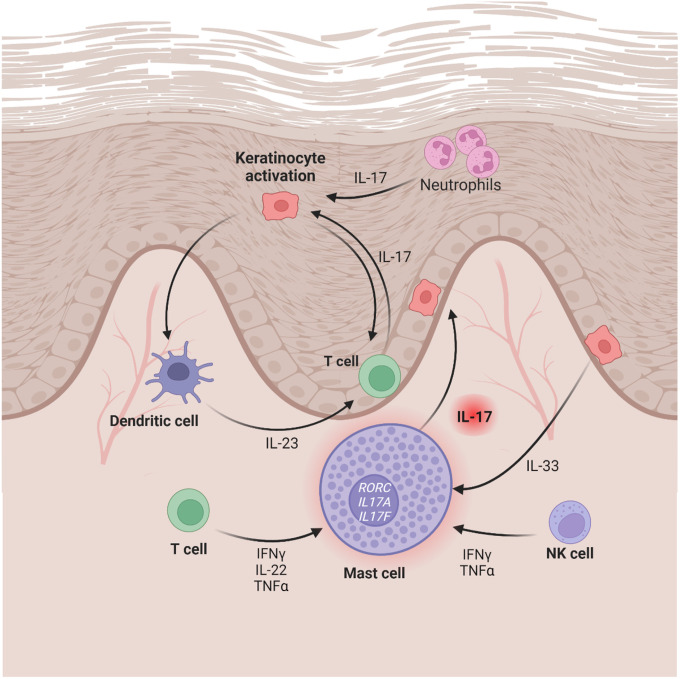
Proposed model for role of mast cells in psoriasis. Mast cells express *RORC, IL17F*, and *IL17A* mRNA, are activated at baseline in psoriatic lesional skin, and produce IL-17. Mast cells might be responsive to various cytokines (e.g. TNFα, IL-22, IFNγ, and IL-33) and, thus, together with T cells, belong to the center of the IL-23/IL-17 axis in psoriasis and might play a crucial role in disease recurrence.Created with BioRender.com.

Little is known about IL-17 expression in the skin during treatment of psoriasis or in clinically resolved lesions. In addition, the actual cellular source of IL-17 in psoriasis has been unclear until now, despite the significance of the cytokine in psoriasis pathogenesis. Using multicolor immunofluorescence staining, a CIBERSORTx analysis of RNA-seq data, and an *in situ* mRNA detection approach, we could carefully study IL-17 expression in psoriasis and in clinically resolved psoriasis after various treatments. This approach enabled us to identify mast cells as an important cellular source of IL-17 (Fig. 7).

In recent decades, it has become clear that mast cells are involved in essential mechanisms of innate and acquired immunity (18, 56). Mast cells are long-lived, tissue-resident cells and, as we and others have shown, they are in close contact with T cells and other cells located in the dermis (19, 57–59). Our findings confirm those of previous studies, showing that the number of mast cells is higher in psoriasis than in healthy skin (16, 60, 61) and non-lesional skin. In active psoriatic lesions, not only T cells, but also neutrophils and mast cells stain positive for IL-17 (13–16). In fact, most IL-17 positive cells in psoriasis are indeed mast cells (62), a finding that we were able to confirm in a cohort of substantial size (n = 23). Unexpectedly, we saw that high numbers of IL-17 + MCs persisted in postlesional skin, irrespective of the anti-psoriatic treatment received (anti-IL-17A, anti-IL-23, UVB or topical dithranol). IL-17 + MCs were located in dense T cell clusters and often close to resident memory T cells (T_RM_) in active psoriasis and resolved lesional skin. MCs and T_RM_ were IL-17 + in active psoriasis, whereas in resolved lesions, T_RM_ were mostly negative for IL-17, while IL-17 + MCs persisted. In areas of clinically resolved psoriasis, T_RM_ serve as a local source of cytokines and have the potential to create a proinflammatory environment that might cause disease recurrence (27, 28). A crosstalk between mast cells and T cells, and specifically T_RM_, has been suggested before in the context of acne (57) and psoriasis (58). However, previous studies placed a focus on T cells as source of cytokines in psoriasis, overlooking the fact that mast cells may produce, store, and release many different mediators that can potentially act in pro-inflammatory ways (19, 20). Our findings suggest that we can now identify mast cells as source of IL-17; and that a MC-T cell interaction is needed to induce cytokine production. This hypothesis is supported by our data showing that MCs isolated from human skin and stimulated with T cell cytokines (TNFα, IFNγ and IL-22) responded by releasing increased amounts of IL-17A.

To our knowledge, *IL17A* mRNA has only ever been detected in human MC progenitor cells isolated from blood by Hueber et al. (63), in the context of acute generalized exanthematous pustulosis and pustular psoriasis in mast cells *in situ* (64), and in a primary human mast cell line by Eliasse et al. (57), who also showed that cell-cell contact of MCs and T cells is needed for *IL17A* expression. Because *IL17A* mRNA has never been detected in mast cells *in vivo*, but only in T cells (13) and neutrophils (55), it was thought that mast cells do not produce the cytokine themselves, but rather take it up and store it in their granules (13, 17, 55). To answer the question whether mast cells have the transcriptional machinery to synthesize IL-17, we used an *in situ* mRNA detection approach with padlock probes specific for transcript variants of *IL17A, IL17F*, and *RORC in* combination with antibody staining of tryptase and CD3. *RORC* encodes the retinoid orphan receptor γt (RORγt), a nuclear hormone receptor, which traditionally is known to program Th17 cell development and function (65, 66). RORγt plays a key role downstream of IL-23, IL-6, and TGFß in the cell lineage specification of uncommitted T-helper cells into Th17 cells (65, 66). It regulates *IL17A* and *IL17F* expression by binding to the conserved non-coding sequence 2 (CNS2) in the *IL77* locus (65, 67). In addition to Th17 cells, other known cell types expressing RORγt are innate lymphoid cells (ILC), γδT cells, and natural killer T (NKT) cells (68–70). In our study we showed that mast cells are positive for *IL17A, IL17F and RORC mRNA* in plaque-type psoriasis; thus, like T cells, these cells have the transcriptional machinery to actively produce IL-17.

The RNA-seq data analysis of resolved psoriatic lesions gave further insights into the therapeutic mechanisms of dithranol. At the molecular levels, these are surprisingly similar to state-of-the-art biologics like anti-IL17A inhibitors. Among the top 20 significantly down-regulated genes after dithranol treatment were well-known genes associated with psoriasis (e.g. genes encoding AMPs, inflammatory cytokines or keratinocyte differentiation genes). These have all been shown to be significantly downregulated with other treatments like biologics (40, 42, 45, 46). While resolved lesions still showed some molecular differences as compared to non-lesional skin, the number of differentially expressed genes was low. We found that *CXCR6* was still upregulated in resolved skin as compared to non-lesional skin. Because this gene is important for the generation and long-term residence of T_RM_ in peripheral tissues (48–51), this finding again suggests that pathogenic resident memory T cells play an important role in psoriasis. However, as our data indicate, skin resident IL-17 + mast cells may be crucial in driving the recurrence of psoriatic disease.

Performing digital flow cytometry by the deconvolution of bulk RNA-seq data allowed us to gain further insights into the immune cell composition of postlesional (psoriatic) skin. While we saw that the levels of activated memory T cells were significantly decreased, we also found that the levels of resting memory T cells slightly increased in resolved psoriatic skin, which might explain why we detected a lingering high expression of *CXCR6*. Furthermore, the number of activated MCs was increased in psoriatic skin, while resting MCs were almost absent, and both returned to normal levels after topical dithranol treatment. These findings highlight the clinical relevance and functional role of MCs in psoriasis treatment. While we saw that IL-17 + MC counts persist in clinically resolved lesions, MCs might stop producing IL-17 at some point during psoriasis treatment and store the cytokine in their granules. Indeed, one study has shown that MCs are able to capture, store, and release bioactive IL-17A (17). It remains unknown however, how long resident MCs survive in the skin or how long they are able to store cytokines.

Other important questions raised by our study are: What triggers psoriasis recurrence? Could IL-17 be stored in mast cells waiting to be released after endogenous and/or exogenous stimulation to reactivate the inflammatory cycle? The alarmin IL-33 could play a role here, as it is secreted by keratinocytes and increased expression has been reported in psoriatic keratinocytes (72, 73). Increased IL-33 expression has also been associated with the Koebner phenomenon (74), which is the appearance of new skin lesions in previously unaffected skin (75). This increase can be triggered by mechanical stimulation or damage to the epidermis (74), and IL-33 can enhance the degranulation of mast cells (76, 77). We hypothesize that this mechanism may also be involved in psoriasis recurrence, whereby IL-33 from keratinocytes may lead to degranulation of MCs and thus the release of IL-17A or de novo production of IL-17A, as our ELISA data with IL-33-stimulated mast cells suggest. Perhaps mast cells could take on the functional role of antigen-presenting cells for pathogenic tissue-resident memory T cells and restart the production of proinflammatory cytokines. Indeed, one study has shown that murine peritoneal mast cells can serve as antigen-presenting cells for T cells (59). In psoriasis, CD4 + T cells express IL-22 when close to mast cells, and mast cells induce IL-22 expression in T cells via IL-6/TNF (58). Our data suggests that mast cells, acting as multifunctional cells, respond not only to T cells but also specifically to T_RM_. However, more studies need to be carried out to determine which signaling pathway leads to increased IL-17 production and how the expression of *RORC* and the further induction of RORγt are regulated in mast cells.

## Methods

### Patients and biopsy samples

Biobank samples were available from previous trials in patients with moderate to severe chronic plaque-type psoriasis treated with biologics, UVB, or topical dithranol. For detailed information about topical dithranol treatment, see (40). In short, biopsy samples were taken from 15 psoriasis patients before (day 0) and at a follow-up visit (4–6 weeks after end of dithranol therapy). At the first visit before starting therapy, one biopsy sample was taken additionally from adjacent non-lesional skin, i.e. at least 5 cm from the edge of the psoriatic skin. For treatment with biologics or UVB, samples were taken in a different study from another group of 15 patients that underwent treatment with anti-IL-17A antibody (ixekizumab), anti-IL-12/23 antibody (ustekinumab), anti-IL-23 antibody (risankizumab or tildrakizumab) or 311 nm UVB. Biopsy samples were taken from lesional psoriatic skin and/or from a clinically resolved lesion at different time points after treatment. For detailed information about these patients and treatments, see supplemental Table S1. One part of each biopsy was fixed in 4% neutral-buffered paraformaldehyde, processed routinely, cut in 3.5 μm sections, and used for histology analyses. The other part was stored for further analysis in RNAlater solution (Invitrogen, California, USA) at −80°C until RNA extraction.

### Immunofluorescence staining and histological evaluation

FFPE sectionsμ (3.5 μM) were deparaffinized and rehydrated and then subjected to antigen retrieval using a pressure cooker and Dako Target Retrieval solution (Dako Agilent, Santa Clara, USA), followed by blocking using Serum-free Dako Protein block (Dako, Agilent, Santa Clara, USA). Primary antibodies were incubated overnight at 4°C and secondary antibodies were incubated for 1h at RT (primary and secondary antibodies are listed in supp. Table S3). To visualize the nuclei, the sections were counterstained with ProLongTM Diamond Anti-fade Mountant with DAPI (Thermo Fisher Scientific, Waltham, USA). Images were obtained with a Nikon A1 confocal microscope and processed using ImageJ software (National Institutes of Health, Bethesda, MD, USA). For quantification of tryptase + mast cells, CD3 + T cells, IL-17 + tryptase + MCs, and IL-17 + CD3 + T cells were counted, all positively/-double-positively stained cells with a visible nucleus in 3–5 randomly selected fields (depending on the size of the tissue) per slide, and the results were averaged to obtain mean cell counts (Fig. 1).

### Culture and stimulation of skin mast cells

Mast cells were isolated from human foreskin tissue as described previously (76–78). To obtain sufficient cell numbers for subsequent culture and experiments, cells from multiple donors were pooled for each mast cell preparation. Skin was obtained from circumcisions, with written informed consent of the patients or their guardians and the approval of the Ethics Committee of the Charite Universitatsmedizin Berlin (protocol code EA1/204/10, March 9, 2018). All experiments were performed in accordance with the principles of the Declaration of Helsinki.

Skin samples were cut into small pieces and incubated with dispase (Boehringer-Mannheim, Mannheim, Germany) at a concentration of 0.5 mg/mL at 4°C overnight. The epidermis was removed, and the dermis was cut into small pieces and digested with 1.5 mg/mL collagenase (Worthington, Lakewood, NJ, USA), 0.75 mg/mL hyaluronidase (Sigma, Deisenhofen, Germany), and 10 μg/mL DNase I (Roche, Basel, Switzerland). Cells were then subsequently filtered through 100μm and 40μm cell strainers. Anti-human c-kit microbeads and an Auto-MACS-separation device were used to further purify the mast cells (both from Miltenyi-Biotec, Bergisch Gladbach, Germany), resulting in 98–100% MC preparations. The purity of the isolated skin mast cells was checked by FACS (double staining for KIT /FcεRI) and acid toluidine blue staining (0.1 % in 0.5 N HCl), as described previously (79, 80). Mast cells were cultured with SCF (100 ng/ml) and IL-4 (20 ng/ml) added freshly twice a week. Stimulation experiments were performed 2–3 days after the last addition of IL-4 and SCF. For stimulation experiments, cells were incubated for 6 hours or 24 hours in complete^™^ Mini EDTA-free protease inhibitor cocktail (Roche, Basel, Switzerland) and recombinant human IL-23 (100 ng/ml), TNFα (10 ng/ml), IL-22 (100 ng/ml), IFNγ (100 ng/ml), and IL-33 (100 ng/ml) were added (all from PeproTech, ThermoFisher Scientific, Waltham, USA).

### IL-17A ELISA

For ELISA, cells in suspension were centrifuged twice at 400 g for 5 min, and the supernatant was stored at −20°C. To measure the IL-17A content, an ELISA MAX^™^ Deluxe Set Human IL-17A (Cat.No: 433914, Biolegend, San Diego, USA) was used as per the manufacturer’s instructions and absorbances were measured at 450 nm on a CLARIOstarPlus photometer (BMG Labtech, Ortenberg, Germany).

### RNA extraction and sequencing

Total RNA was extracted from frozen skin biopsies of psoriasis patients. To facilitate homogenization, tissues were cut in 20 μm sections using a cryomicrotome and collected in precooled MagNA Lyser Green Beads tubes (Roche, Basel, Switzerland). Further disruption of tissue was performed with a MagNA Lyser Instrument (Roche, Basel, Switzerland). After efficient homogenization, total RNA was extracted using the miRNeasy Mini Kit (Qiagen, Hilden, Germany), according to the manufacturer’s instructions. To extract RNA from the cultured mast cells, cell supernatants were removed, and cell pellets were lysed in Qiazol lysis reagent; RNA was then isolated from the pellet using the miRNeasy Mini Kit (Qiagen, Hilden, Germany), according to the manufacturer’s instructions. To ensure complete DNA removal, an on-column DNase digestion was performed, and RNA was eluted in 15–20 μl of RNase-free water. The RNA concentration was determined with a NanoDrop^™^ 2000 spectrophotometer (Thermo Fisher Scientific, Waltham, USA) and its quality was confirmed with a BioAnalyzer BA2100 (Agilent, Foster City, CA, USA). The quality of all RNA samples was between RIN 5 and 8. rRNA was eliminated by using the NEBnext rRNA Depletion Kit (Human/Mouse/Rat) with beads (New England Biolabs; Ipswich, MA, USA) according to the manufacturer’s instructions. Library preparation was done with the NEBnext Ultra II Directional RNA Library Prep Kit for Illumina Kit with beads (New England Biolabs; Ipswich, MA, USA). To identify the samples after sequencing, they were labelled with the NEBNext Multiplex Oligos, Set 1 (New England Biolabs; Ipswich, MA, USA) and NEBNext Multiplex Oligos, Set 2 (New England Biolabs; Ipswich, MA, USA). The final libraries were then quantified with a Quantus Fluorometer (Promega; Madison, WI, USA) using the QuantiFluor ONE dsDNA System (Promega; Madison, WI, USA). To check the fragment sizes of the library, all samples were measured with a Agilent 2100 Bioanalyzer using the DNA High Sensitivity LabChip (Agilent; Foster City, CA, USA). A mean peak size of 417 bp was observed. Vienna BioCenter Core Facilities GmbH (VBCF; Vienna, Austria) sequenced the equimolar pooled samples on an Illumina NovaSeq SP in PE150 mode (Illumina; San Diego, CA) after performing an additional 0.9X bead purification with SPRIselect^™^ beads (Beckman Coulter; Brea, CA) according to the manufacturer’s instructions. After a demultiplexing step, the FASTQ files were used for data analysis.

### RNA sequencing – data analysis

The paired-end raw sequencing data were imported into a private Galaxy (v23.0.1) instance running on the MedBioNode cluster from the Medical University Graz. After an initial quality control step was performed with FastQC (v0.73) and MultiQC (v1.11), the reads were trimmed with the Trimmomatic program (v0.38.1, minimum length 40 bp, and removing Illumina-specific adaptor sequences). Reads were aligned against the reference genome GRCh38.p13 by using the Ensembl gene annotation Homo_sapiens.GRCh38.108.gtf with RNASTAR (v2.78)(81) in two-pass mode with standard settings, except for --outFilterType YES, --outFilterMismatchNoverLmax 0.05, --outFilterMultimapNmax 5, --alignSJoverhangMin 8 in the second pass, and HTSeq framework (v0.9.1) was used to generate count data (average per sample: 22 Mio). The statistical analysis and visualization were performed with TIBCO Spotfire (v12.5, Palo Alto, USA) or in R (4.2.1) with RStudio Desktop (2023.06.0) using the packages: RColorBrewer, readxl, openxlsx, tibble, tidyr, dplyr, ashr, ggplot2, colorspace, ggforce, scales, ggrepel, pheatmap, and DESeq2 (82, 83)

Out of the 62,703 annotated genes, a total of 40,435 low-count genes were filtered out either because the median across all samples was below 10 counts or no group had a non-zero count in at least in 90% of the samples with a group median count >10.

To determine the differential gene expression, the model *PatientJD + group* and Benjamini-Hochberg (BH) procedure with multiple test adjustment were used (cut-off-adjusted pvalue < 0.05), since the addition of *Patient_JD* was found to be significant for most differentially expressed genes (DEG). Furthermore, fold changes were shrunk with adaptive shrinkage (ashr)(84). Shrunken log_2_ fold changes (*log*_*2*_ *(ashr fold changes))* were abbreviated LFC, and only stable LFCs were considered for significance testing (s-value <0.005). Principal component analysis (PCA) was performed with the top 500 genes that had the highest row variance on variance stabilizing transformed (VST) data. Counts, VST, and DEG results are provided in Supplementary Data File 2.

Gene set enrichment analysis (GSEA) was performed in *clusterProfiler::compareCluster()* R using a gene ontology (GO) database for biological process (BP) based on LFC. The minimal gene set size was set to 25, the maximum to 500 and the p-values were adjusted by using the Benjamini-Hochberg (BH) procedure and setting a p-value cutoff of 0.05. All GSEA results are provided in Supplementary Data File 2.

When performing digital flow cytometry with CIBERSORTx (53), the genes (after filtration) were normalized to transcripts per million (TPM). The 427 genes with a median count of over 10 in all samples matching the LM22 reference matrix genes were uploaded (https://cibersortx.stanford.edu/). Deconvolution was performed by applying batch correction in the B-mode, and 500 permutations were made, estimating the scaled absolute abundance of 22 immune cell types. Cell types with at least 75% of non-zero estimates across all samples or at least 90% of non-zero estimates in at least one group were statistically analysed by using a linear mixed models (LMM) with a model equivalent to that used in the DEG analysis: *~group + 1/PatienUD*.

In order to compare RNA-seq data from psoriasis patients treated with topical dithranol to RNA-seq data from anti-IL-17A-treated psoriasis patients (supp. Figure 3), gene counts from Liu et al. 2022 (52) were downloaded from Gene Expression Omnibus (GEO; dataset GSE171012, NCBI, NIH, Bethesda, USA) and re-analysed in the same way as described above. Out of the 57,947 annotated genes, a total of 37,756 low-count genes were filtered out. Thereof, 17,420 genes were found in both data sets and directly compared in terms of their significance and LFC.

### In situ mRNA detection with padlock probes

*In situ* detection of mRNA using padlock probe technology was used to localize *IL17A, IL17F*, and *RORC* mRNA transcripts as described previously (85, 86). Padlock probes consist of oligonucleotides that hybridize specifically to the target sequence (e.g. *IL17A, IL17F*, and *RORC)*. After hybridization, the padlock probe is ligated, creating a DNA circle. This DNA circle is then amplified by performing rolling circle amplification (RCA). The RCA products can be detected with fluorescent detection probes, resulting in the generation of bright fluorescent signals for each specifically targeted transcript (e.g. *IL17A* in CY3 and *IL17F* in Texas Red). Oligonucleotides and padlock probes were designed to capture the exon sequences of the mRNA transcript variants of *IL17A, IL17F*, and *RORC* by using freely available Python software for the padlock probe design (87). Oligonucleotides were ordered from Integrated DNA Technologies (Leuven, Belgium). Tryptase and CD3 staining were performed according to the *in situ* protocol. First, a wash step with PBS was performed, followed by a seven-minute blocking step with Ultra Vision Protein Block (Epredia). Then, the primary antibodies were incubated for 30 minutes at RT and the slides were then washed again with PBS. Secondary antibodies were then applied (goat anti-mouse Secondary Antibody Alexa Fluor^™^ 633, Thermo Fisher, dilution 1:200 in PBS and goat anti-rabbit Secondary Antibody Alexa Fluor^™^ 555 1:200 in PBS) and incubated for 30 minutes at RT. Imaging was performed using a digital slide scanner (Olympus SLIDEVIEW VS200) with a 40x objective. Oligonucleotide sequences can be found in the supplemental data (supp. Table S2).

### Statistical analyses

As appropriate, the Shapiro-Wilk test, Mann-Whitney *U*test, paired or unpaired t-tests, Wilcoxon signed-rank test, Spearman correlation, Friedman or Kruskal-Wallis test combined with Dunn’s multiple comparisons test were carried out using GraphPad Prism version 10 (GraphPad software, California, USA) to test for normality, perform a correlation analysis, or to compare different groups, respectively. Significance was set at a *p*-value of < 0.05.

### Study approval

Clinical data and samples from psoriasis patients were available from different studies. All clinical trial procedures were approved by the Ethics Committee of the Medical University of Graz (protocol number 26–540 ex 13/14 1370–2014) or the federal state of Carinthia, Austria (protocol number A23/15). Skin samples for mast cell isolation were obtained from circumcisions, with the approval of the Ethics Committee of the Charite Universitätsmedizin Berlin (protocol code EA1/204/10, March 9, 2018). All participants gave their written informed consent in accordance with the principles of the Declaration of Helsinki.

## Data Availability

All data needed to evaluate the conclusions of the current manuscript are reported in the paper, Supplementary data files or are accessible through GEO Series GSE249936 (https://www.ncbi.nlm.nih.gov/geo/query/acc.cgi?acc=GSE249936).
